# Ten-year National Trends in Patient Characteristics and 30-day Outcomes of Distal Radius Fracture Open Reduction and Internal Fixation

**DOI:** 10.5435/JAAOSGlobal-D-22-00181

**Published:** 2022-09-22

**Authors:** Dafang Zhang, George S. M. Dyer, Brandon E. Earp, Philip Blazar

**Affiliations:** From the Department of Orthopaedic Surgery, Brigham and Women's Hospital, Boston, MA (Dr. Zhang, Dr. Dyer, Dr. Earp, and Dr. Blazar), and Harvard Medical School, Boston, MA (Dr. Zhang, Dr. Dyer, Dr. Earp, and Dr. Blazar).

## Abstract

Despite increased utilization, recent temporal trends in patient comorbidities and episode-of-care outcomes for distal radius fracture open reduction and internal fixation (ORIF) have not been studied. The objective of this study was to assess 10-year national trends in (1) patient characteristics and comorbidities and (2) 30-day postoperative adverse outcomes for patients who underwent distal radius fracture ORIF using a large patient database.

**Methods:**

A retrospective study was conducted using the National Surgical Quality Improvement Program database by querying the procedural codes for distal radius fracture ORIF from January 1, 2011, to December 31, 2020. A cohort of 28,616 adult patients who underwent distal radius fracture ORIF during the study period was included. Baseline patient characteristics and 30-day postoperative outcome data were collected for each year of the study. Temporal trends from 2011 to 2020 for all variables were assessed using the two-tailed Mann-Kendall trend test.

**Results:**

Of the 28,616 patients in the study cohort, the mean age was 56.4 years and 73.1% were female. Demographically, there was a trend toward higher body mass index, higher American Society of Anesthesiologists classification, and lower proportions of active smokers; functionally dependent patients; and patients with hypertension, chronic obstructive pulmonary disease, and bleeding disorder. There was a trend toward worse sepsis physiology and wound classification on presentation. There was a trend toward fewer blood transfusions, hospital readmissions, and revision surgeries; however, there was an increasing trend for the development of a superficial surgical site infection.

**Discussion:**

Ten-year national trends in distal radius fracture ORIF demonstrated improvements in several important patient comorbidities and the rates of readmission and revision surgery. However, overall patient comorbidities, sepsis physiology, and wound classification have worsened, and the rate of superficial surgical site infections has increased. Trends in patient comorbidities and episode-of-care outcomes should be considered when transitioning toward value-based care models.

Distal radius fractures are the most common upper extremity fractures and occur with a frequency of 16.2 per 10,000 people.^1^ The incidence of distal radius fractures is increasing across all age groups globally as is the utilization of open reduction and internal fixation (ORIF) for the treatment of this fracture.^1,2,3,4,5,6^ The increasing rate of distal radius fracture ORIF has been largely propelled by the advent of volar locking plate technology, which enables angular stable control of articular fragments, allowing for early mobilization.^7^

Although the injury and treatment are common, distal radius fractures are associated with notable patient morbidity and mortality. Patients with a distal radius fracture have a 14% higher rate of mortality at 7 years after injury compared with the general population, which likely reflects underlying patient comorbidities and frailty.^8^ With surgical treatment of distal radius fractures, the 90-day complication rate is estimated at 4% and the 1-year complication rate is estimated at more than 30%.^9^ Distal radius fracture treatment and its associated complications continue to be a source of economic burden on the healthcare system.^10,11^ This is of growing importance as institutions transition toward value-based care initiatives that balance quality and outcomes against costs of care.^12^

Understanding temporal trends in patient comorbidities and risk factors and episode-of-care outcomes can offer insights into the value of treatments rendered to patients, inform patient counseling, and identify areas for future improvement. The objective of this study was to assess 10-year national trends in patients who undergo distal radius fracture ORIF using a large patient database. Specifically, we aimed to assess temporal trends in (1) patient characteristics and comorbidities and (2) 30-day postoperative adverse outcomes for patients who underwent distal radius fracture ORIF from 2011 to 2020.

## Methods

### Study Design and Patient Identification

A retrospective study was conducted to assess temporal trends in patient characteristics and 30-day postoperative outcomes of distal radius fracture ORIF using the American College of Surgeons (ACS) National Surgical Quality Improvement Program (NSQIP) database. NSQIP is a validated, risk-adjusted database of patients 18 years or older undergoing major surgery in over 700 US-based hospitals. The database collects preoperative patient characteristics and 30-day postoperative outcomes, such as complications, readmission, revision surgery, and mortality. The data are acquired by medical chart review and confirmed by written or telephone patient contact at the end of the 30-day postoperative period; the accuracy of the data is ensured through random audits and clinical reviews.^13^

The NSQIP database was used to identify patients who underwent distal radius fracture ORIF from January 1, 2011, through December 31, 2020, by querying Current Procedural Terminology codes 25607 (open treatment of distal radial extra-articular fracture or epiphyseal separation, with internal fixation), 25608 (open treatment of distal radial intra-articular fracture or epiphyseal separation; with internal fixation of 2 fragments), and 25609 (open treatment of distal radial intra-articular fracture or epiphyseal separation; with internal fixation of 3 or more fragments). The query resulted in 28,616 adult patients who underwent distal radius fracture ORIF during the study period.

### Study Variables

Baseline patient characteristics and 30-day postoperative outcome data were collected for each year of the study, from 2011 to 2020. The following patient-related variables were analyzed: age, sex, body mass index (BMI), American Society of Anesthesiologists (ASA) classification, diabetes mellitus, current smoking status, functional status (independent, partial dependent, or totally dependent), dyspnea (none, with moderate exertion, or at rest), chronic obstructive pulmonary disease (COPD), ascites, congestive heart failure (CHF), hypertension requiring medication, renal failure requiring dialysis, disseminated cancer, chronic steroid or immunosuppressive therapy, malnourishment, and bleeding disorder. ACS NSQIP provides detailed definitions for each medical comorbidity, which may be referenced.^13^ The following injury-related and treatment-related variables were analyzed: surgical time (minutes), sepsis physiology (none, systemic inflammatory response syndrome, sepsis, or septic shock), and surgical wound classification (clean, clean/contaminated, contaminated, or dirty/infected).

The following 30-day postoperative outcomes were analyzed: superficial surgical site infection, deep surgical site infection, organ/space surgical site infection, wound dehiscence, pneumonia, unplanned reintubation, pulmonary embolism, persistent ventilator requirement, progressive renal insufficiency, urinary tract infection, stroke, cardiac arrest, myocardial infarction, blood transfusion, deep vein thrombosis, septic shock, hospital readmission, revision surgery, and death. Superficial surgical site infection, deep surgical site infection, and organ/space surgical site infection are defined by ACS NSQIP in accordance with the Centers for Disease Control and Prevention (CDC).^14^ ACS NSQIP provides detailed definitions for 30-day complications, which may be referenced.^13^

### Statistical Analysis

Descriptive statistics for the study cohort were calculated. Means and SDs for parametric continuous variables, medians and interquartile ranges for nonparametric continuous variables, and percentages for categorical variables were calculated. All variables had greater than 95% data completeness, and statistical analyses were conducted using complete data sets only. Temporal trends from 2011 to 2020 for all variables were assessed using the 2-tailed Mann-Kendall trend test. Kendall τ coefficients and *P* values were calculated. Kendall τ coefficient is a measure of relationship between nonparametric ranked data, such as a value of 1 represents a perfect relationship, a value of 0 represents no relationship, and a value of −1 represents a perfect inverse relationship. The standard significance criterion of α = 0.05 was used, and temporal trends were assigned as increasing, no trend, or decreasing at the standard 95% level of confidence. Data preparation and statistical analysis were done using R and SAS.

## Results

A final cohort of 28,616 patients who underwent distal radius fracture ORIF from 2011 to 2020 was identified using the NSQIP database and included in this study. The baseline characteristics of the study cohort are summarized in Table 1.

**Table 1:** Patient Characteristics of the Overall Study Cohort, Patients From Year 2011, and Patients From Year 2020

Factor	Overall cohort	2011	2020	Kendall τ^a^	*P* Value	Temporal trend^b^
No. of patients (n)	28,616	805	4,844	0.996	0.0001	Increasing
	Mean (SD)	Mean (SD)	Mean (SD)			
Age (yr)	56.4 (16.4)	56.1 (16.8)	56.2 (16.4)	−0.003	0.5	No trend
	Median (IQR)	Median (IQR)	Median (IQR)			
BMI	27.1 (23.6-31.6)	23.2 (26.5-30.7)	27.2 (23.8-31.8)	0.014	0.0009	Increasing
Surgical time (min)	67 (50-92)	69 (52-95)	68 (50-93)	−0.012	0.004	Decreasing
	n (%)	n (%)	n (%)			
Male sex	7,709 (26.9)	230 (28.6)	1,306 (27.0)	0.002	0.7	No trend
ASA classification				0.025	< 0.0001	Increasing
1	5,083 (17.8)	173 (21.5)	445 (1.4)			
2	15,727 (55.0)	441 (54.8)	13,136 (41.4)			
3	7,313 (25.6)	180 (22.4)	17,174 (54.3)			
4	452 (1.6)	11 (1.4)	938 (3.0)			
Diabetes mellitus	2,385 (8.3)	63 (7.8)	386 (8.0)	−0.005	0.3	No trend
Current smoker	5,140 (18.0)	156 (19.4)	819 (16.9)	−0.018	0.0006	Decreasing
COPD	964 (3.4)	28 (3.5)	138 (2.9)	−0.013	0.01	Decreasing
Ascites	9 (0.0)	0 (0.0)	0 (0.0)	−0.007	0.2	No trend
CHF	88 (0.3)	1 (0.1)	12 (0.3)	0.002	0.7	No trend
Hypertension	9,129 (31.9)	279 (34.7)	1,441 (29.8)	−0.021	< 0.0001	Decreasing
Dialysis	70 (0.2)	0 (0.0)	12 (0.3)	0.00002	0.9	No trend
Disseminated cancer	64 (0.2)	5 (0.6)	8 (0.2)	−0.004	0.5	No trend
Steroids/immunosuppression	621 (2.2)	13 (1.6)	107 (2.2)	0.009	0.09	No trend
Malnourishment	50 (0.2)	1 (0.1)	12 (0.3)	0.004	0.4	No trend
Bleeding disorder	653 (2.3)	25 (3.1)	89 (1.8)	−0.021	< 0.0001	Decreasing
Dyspnea				−0.009	0.08	No trend
None	27,847 (97.3)	773 (96.0)	4,714 (97.3)			
Moderate exertion	702 (2.5)	31 (3.9)	122 (2.5)			
At rest	67 (0.2)	1 (0.1)	8 (0.2)			
Functional dependency				−0.018	0.0003	Decreasing
Independent	27,722 (98.4)	773 (96.9)	4,707 (97.3)			
Partially dependent	419 (1.5)	23 (2.9)	56 (1.2)			
Totally dependent	32 (0.1)	2 (0.3)	9 (0.2)			
Sepsis physiology				0.012	0.02	Increasing
None	28,289 (98.9)	798 (99.1)	4,777 (98.6)			
SIRS	320 (1.1)	7 (0.9)	66 (1.4)			
Sepsis	6 (0.0)	0 (0.0)	1 (0.0)			
Wound classification				0.015	0.004	Increasing
Clean	27,603 (96.5)	783 (97.3)	4,635 (95.7)			
Clean/contaminated	495 (1.7)	10 (1.2)	104 (2.2)			
Contaminated	373 (1.3)	10 (1.2)	73 (1.5)			
Dirty/infected	145 (0.5)	2 (0.3)	32 (0.7)			

ASA = American Society of Anesthesiologists, BMI = body mass index, CHF, congestive heart failure, COPD = chronic obstructive pulmonary disease, SIRS = systemic inflammatory response syndrome

Kendall τ coefficients, *P* values, and temporal trends are shown.

a Kendall τ and associated *P* values are calculated for the 10-year time frame from 2011 to 2020 to assess for monotonic temporal trends across the years of the study period.

b Temporal trends are assigned at the 95% level of confidence.

### Trends in Patient Characteristics

**Figure 1:**
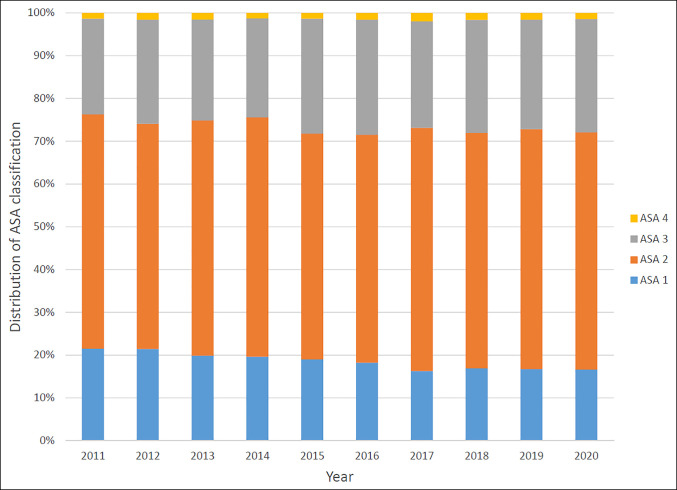
Stacked bar graph illustrating the distribution of ASA classification among patients who underwent distal radius fracture open reduction and internal fixation from 2011 to 2020. ASA = American Society of Anesthesiologists.

Among patients who underwent distal radius fracture ORIF from 2011 to 2020, there was an increasing temporal trend toward higher BMI (τ = 0.014, *P* = 0.0009) and higher ASA classification (τ = 0.025, *P* < 0.0001) (Figure 1). An increasing temporal trend was observed toward presentation with more severe sepsis physiology (τ = 0.012, *P* = 0.02) and with worse surgical wound classification (τ = 0.015, *P* = 0.004). There was a decreasing temporal trend in the proportion of patients with an active smoking history (τ = −0.018, *P* = 0.0006), COPD (τ = −0.013, *P* = 0.001), hypertension (τ = −0.021, *P* < 0.0001), and bleeding disorder (τ = −0.021, *P* < 0.0001). There was a decreasing temporal trend in the proportion of patients who were more functionally dependent (τ = −0.018, *P* = 0.0003). The median surgical time showed a decreasing trend from 2011 to 2020 (τ = −0.012, *P* = 0.004). There was no significant temporal trend in age; sex; or proportion of patients with dyspnea, diabetes mellitus, ascites, CHF, renal failure on dialysis, disseminated cancer, chronic steroid or immunosuppressive use, or malnourishment (Table 1).

### Trends in 30-Day Postoperative Outcomes

**Figure 2:**
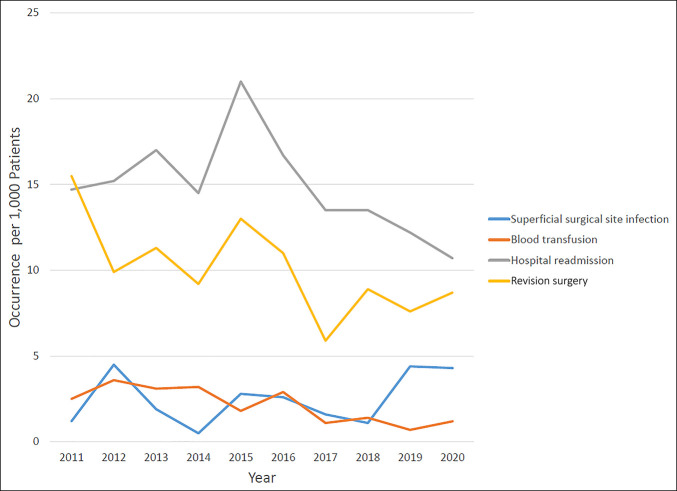
Line graph illustrating the occurrence of superficial surgical site infection, blood transfusion, hospital readmission, and revision surgery per 1,000 patients from 2011 to 2020.

Among patients who underwent distal radius fracture ORIF from 2011 to 2020, there was a decreasing temporal trend for the proportion of patients who underwent blood transfusion (τ = −0.015, *P* = 0.003), hospital readmission (τ = −0.016, *P* = 0.001), and revision surgery (τ = −0.011, *P* = 0.02). However, there was an increasing temporal trend for the proportion of patients who developed a superficial surgical site infection (τ = 0.013, *P* = 0.01) (Figure 2). No significant temporal trend was observed in the proportion of patients with deep surgical site infection, organ/space surgical site infection, wound dehiscence, pneumonia, unplanned reintubation, pulmonary embolism, persistent ventilator requirement, renal insufficiency, urinary tract infection, stroke, cardiac arrest, myocardial infarction, deep vein thrombosis, septic shock, or death (Table 2).

**Table 2:** Thirty-Day Postoperative Outcomes of the Overall Study Cohort, Patients From Year 2011, and Patients From Year 2020

Factor	Overall cohort n (%)	2011 n (%)	2020 n (%)	Kendall τ^a^	*P* Value	Temporal trend^b^
Superficial surgical site infection	77 (0.27)	1 (0.12)	21 (0.43)	0.013	0.01	Increasing
Deep surgical site infection	13 (0.05)	0 (0.00)	2 (0.04)	−0.002	0.7	No trend
Organ/space surgical site infection	12 (0.04)	1 (0.12)	2 (0.04)	0.001	0.9	No trend
Wound dehiscence	13 (0.05)	0 (0.00)	3 (0.06)	0.008	0.1	No trend
Pneumonia	41 (0.14)	1 (0.12)	7 (0.14)	−0.0001	0.9	No trend
Unplanned reintubation	16 (0.06)	0 (0.00)	3 (0.06)	0.0009	0.9	No trend
Pulmonary embolism	14 (0.05)	0 (0.00)	1 (0.02)	0.006	0.2	No trend
Ventilator requirement	5 (0.02)	0 (0.00)	0 (0.00)	−0.0003	0.9	No trend
Renal insufficiency	12 (0.04)	0 (0.00)	1 (0.02)	−0.001	0.8	No trend
Urinary tract infection	92 (0.32)	1 (0.12)	18 (0.37)	0.002	0.7	No trend
Stroke	9 (0.03)	0 (0.00)	1 (0.02)	−0.003	0.5	No trend
Cardiac arrest	9 (0.03)	0 (0.00)	4 (0.08)	0.007	0.2	No trend
Myocardial infarction	14 (0.05)	1 (0.12)	5 (0.10)	0.006	0.2	No trend
Blood transfusion	51 (0.18)	2 (0.25)	6 (0.12)	−0.015	0.003	Decreasing
Deep vein thrombosis	16 (0.06)	0 (0.00)	3 (0.06)	0.002	0.7	No trend
Septic shock	8 (0.03)	0 (0.00)	1 (0.02)	−0.002	0.6	No trend
Readmission	408 (1.43)	10 (1.47)	52 (1.07)	−0.016	0.001	Decreasing
Revision surgery	266 (0.93)	12 (1.55)	42 (0.87)	−0.011	0.02	Decreasing
Death	38 (0.13)	2 (0.25)	8 (0.17)	−0.003	0.6	No trend

Kendall τ coefficients, *P* values, and temporal trends are shown.

a Kendall τ and associated *P* values are calculated for the 10-year time frame from 2011 to 2020 to assess for monotonic temporal trends across the years of the study period.

b Temporal trends are assigned at the 95% level of confidence.

## Discussion

We have demonstrated notable temporal trends in comorbidities and episode-of-care outcomes in patients who underwent distal radius fracture ORIF from 2011 to 2020 using the NSQIP database. Trends in patient comorbidities were mixed. Although the proportions of specific comorbidities, such as COPD, hypertension, bleeding disorders, and smoking, have improved over the 10-year study period, patients seem to have more medical comorbidities overall, have higher BMI, present with more severe sepsis physiology, and present with worse surgical wound classification. Trends in a number of episode-of-care metrics showed improvement during the 10-year study period, including shorter surgical times, fewer blood transfusions, fewer hospital readmissions, and fewer revision surgeries. However, our findings showed an increasing trend of superficial surgical site infections.

The improvements in episode-of-care outcomes after distal radius fracture ORIF that we observed likely resulted from a combination of factors. First, we observed improvements in important baseline patient characteristics during the 10-year study period, including comorbid hypertension, COPD, and smoking, which likely translated to improved outcomes. Data from the CDC have shown that the prevalence of smoking among the general US population has decreased during the study period, likely because of growing public awareness of the harms of smoking, evidence for the rapid benefits of smoking cessation, and financial incentives from tobacco-related excise taxes.^15^ However, the prevalence of COPD^16^ and hypertension^17^ has steadily increased in the general US population during the study period, and lower rates of these comorbidities in the distal radius fracture ORIF population may represent injury-specific demographic differences or patient selection for ORIF. In a previous database study of distal radius fractures, Mosenthal et al ^18^ showed that hypertension, COPD, and smoking were independently associated with complications after surgical treatment, with hypertension having the strongest correlation. In a database study of 16,158 patients who underwent distal radius fracture ORIF, Galivanche et al ^19^ showed that smoking was independently associated with 30-day postoperative complications among propensity score-matched cohorts. Moreover, smoking has been associated with 30-day hospital readmission after outpatient elective hand and elbow surgery.^20^ Finally, there has been evidence that patient frailty is associated with 30-day complications, readmission, and revision surgery after distal radius fracture ORIF. Frailty is often measured by the 5-item modified frailty index, which takes into account hypertension, COPD, functional dependency, CHF, and diabetes mellitus.^21^ Improvements in these key patient comorbidities from 2011 to 2020 may have contributed to improvements in readmissions and revision surgeries, and fewer patients with baseline bleeding disorders may have contributed to fewer blood transfusion requirements in our study. A second potential explanation for the improving temporal trends in readmissions and revision surgeries is that surgeon technique, experience, and expertise may have evolved and improved with time. This is reflected by a trend toward shorter surgical time, although not clinically significant. During the 10-year study period, there has been a growing shift toward volar locking plate fixation of these fractures,^22^ leading to refinement of the surgical technique and technology and awareness of potential pitfalls such as plate prominence,^23^ all of which may have contributed to improved outcomes.

Our findings have identified areas for future improvement. Although the proportions of specific key comorbidities have improved, it seems that patients treated with distal radius fracture ORIF have overall more comorbidities, as evidenced by a rising ASA classification across the 10-year study period. Moreover, patients are trending toward a higher BMI, which has been associated with 30-day complications and revision surgery after distal radius fracture ORIF.^24^ During the same period, data from CDC show increasing average BMI in the general US population, suggesting that we are treating an increasing proportion of obese patients over the past decade.^25^ We observed a trend toward higher proportions of presentation with sepsis physiology and more severe wound classifications among patients undergoing distal radius fracture ORIF. This effect may be related to the growing utilization and expanding indications of volar locking plate fixation. Although it is outside the scope of this study, we speculate that in previous years, some of these patients would have been treated closed or with external fixation and, therefore, would not have been captured by the procedural codes for distal radius fracture ORIF. In addition, we speculate that patients presenting with less severe wound classifications and in less physiologic distress are increasingly preferentially treated in ambulatory surgical settings rather than hospital settings, funneling them out of the NSQIP database. During the 10-year study period, we have found an increasing trend for the development of a superficial surgical site infection. This may be related to patient selection because ASA classification, wound classification, and obesity have been clearly and independently associated with the development of surgical site infection after surgery generally and orthopaedic trauma surgery specifically.^26-28^ Awareness of this adverse temporal trend may better inform patient selection and prognostication and improve patient preoperative counseling regarding the risks of surgery.

There are several limitations of this study, some of which are inherent to the use of a large patient database and to this database in particular. First, the findings of a retrospective analysis of a large database lack granularity. In the current treatment landscape, we estimate that more than 80% of distal radius fractures are treated with volar locking plate fixation alone^22^; however, the Current Procedural Terminology codes used for this study are unable to further differentiate between various ORIF techniques, including dorsal plating, fragment-specific fixation, or wrist-spanning bridge plating. We have been unable to study surgeon technique, duration of immobilization, or rehabilitation protocols. Because the NSQIP database collects only specific comorbidities, we assessed medical comorbidities individually rather in aggregate with indices such as the Charlson Comorbidity Index or the Elixhauser Comorbidity Index. Furthermore, the NSQIP database lacks the granularity necessary to robustly identify cases of polytrauma, which may be especially germane to the subset of patients who presented with sepsis physiology. Second, the NSQIP database collects postoperative outcome data up to 30 days after surgery. Adverse events outside this time frame are outside the scope of this study. Third, the NSQIP database includes hospitals but not ambulatory surgery centers, where distal radius fracture ORIF are commonly done. Our findings are vulnerable to selection bias if patients with fewer comorbidities are preferentially treated at ambulatory surgery centers rather than hospitals over time; we would expect such a bias to overestimate the true proportions of comorbidities and 30-day adverse events among distal radius fracture ORIF patients. Fourth, our study focused only on distal radius ORIF, and therefore, we are unable to comment on trends in the prevalence, comorbidities, and outcomes of patients who undergo other treatments for distal radius fractures, such as casting or percutaneous fixation.

This study of the 10-year national trends in distal radius fracture ORIF demonstrated notable improvements in several important patient comorbidities. From 2011 to 2020, there has been a decreasing temporal trend for hospital readmission and revision surgery after distal radius fracture ORIF. However, there has been a worsening trend in the overall medical comorbidities, physiologic distress, and surgical wound classification of distal radius fracture ORIF patients during this period, and the rate of superficial surgical site infections has worsened. Knowledge of this trend in patient selection and associated adverse events may influence patient selection for surgery and inform preoperative counseling about the risks of surgery. Moreover, as institutions transition toward value-based care models that weigh quality and outcomes against costs of care, it is important to consider changes in patient characteristics insofar because they directly affect episode-of-care outcomes and costs.
